# Exploration of Potential miRNA Biomarkers and Prediction for Ovarian Cancer Using Artificial Intelligence

**DOI:** 10.3389/fgene.2021.724785

**Published:** 2021-11-25

**Authors:** Farzaneh Hamidi, Neda Gilani, Reza Arabi Belaghi, Parvin Sarbakhsh, Tuba Edgünlü, Pasqualina Santaguida

**Affiliations:** ^1^ Department of Statistics and Epidemiology, Faculty of Health, Tabriz University of Medical Sciences, Tabriz, Iran; ^2^ Department of Statistics, Faculty of Mathematical Science, University of Tabriz, Tabriz, Iran; ^3^ Department of Mathematics, Applied Mathematics and Statistics, Uppsala University, Uppsala, Sweden; ^4^ Department of Medical Biology, Faculty of Medicine, Muğla Sıtkı Koçman University, Muğla, Turkey; ^5^ Department of Health Research and Methods, McMaster University, Hamilton, ON, Canada

**Keywords:** Biomarker, Elasticnet, Feature Selection, Gene Expression Omnibus (GEO), Lasso, Machine Learning, Ovarian Cancer

## Abstract

Ovarian cancer is the second most dangerous gynecologic cancer with a high mortality rate. The classification of gene expression data from high-dimensional and small-sample gene expression data is a challenging task. The discovery of miRNAs, a small non-coding RNA with 18–25 nucleotides in length that regulates gene expression, has revealed the existence of a new array for regulation of genes and has been reported as playing a serious role in cancer. By using LASSO and Elastic Net as embedded algorithms of feature selection techniques, the present study identified 10 miRNAs that were regulated in ovarian serum cancer samples compared to non-cancer samples in public available dataset GSE106817: hsa-miR-5100, hsa-miR-6800-5p, hsa-miR-1233-5p, hsa-miR-4532, hsa-miR-4783-3p, hsa-miR-4787-3p, hsa-miR-1228-5p, hsa-miR-1290, hsa-miR-3184-5p, and hsa-miR-320b. Further, we implemented state-of-the-art machine learning classifiers, such as logistic regression, random forest, artificial neural network, XGBoost, and decision trees to build clinical prediction models. Next, the diagnostic performance of these models with identified miRNAs was evaluated in the internal (GSE106817) and external validation dataset (GSE113486) by ROC analysis. The results showed that first four prediction models consistently yielded an AUC of 100%. Our findings provide significant evidence that the serum miRNA profile represents a promising diagnostic biomarker for ovarian cancer.

## Introduction

Ovarian cancer is a major clinical challenge in gynecologic oncology. Due to the lack of a proper biomarker-based screening method, most patients are asymptomatic until the disease has metastasized and two-thirds of patients are diagnosed with advanced stages (Lheureux et al., 2019). The International Federation of Gynecology and Obstetrics (FIGO) reported that in the majority of those diagnosed in stage three or four ovarian cancer (2014), more than 70% will have a relapse of their disease within the first 5 years (Reid et al., 2017). Currently, there is an acute need to know potential biomarkers that could lead to the growth of modern and more accurate predictors for ovarian cancer diagnosis and prognosis. As noted, one of the most common gynecologic malignancy is epithelial ovarian cancer (EOC), with each year of about 230,000 new cases and almost 140,000 deaths (Greenlee et al., 2001). In 2020, it is estimated that approximately 21,750 new cases and 13,940 deaths occurred in the United States and 29,000 deaths happened in Europe due to ovarian cancer (Iorio et al., 2007). Therefore, the underlying molecular mechanism has not yet been elucidated. The timely prediction of ovarian cancer would benefit women, healthcare systems, and society as a whole. Accurate and reliable prediction models would enable preventative interventions to reduce the morbidity and mortality associated with ovarian cancer (Harter et al., 2008).

### MicroRNAs

MicroRNAs (miRNA) are important genomic datasets in the human genome that play a regulative impress in cellular processes. miRNAs are a type of non-coding RNA with 18–25 nucleotides in length and reported to play a serious role in human cancers. miRNAs are often copied from DNA sequences to primary miRNAs. Subsequent processes lead to the production of precursor miRNAs and mature miRNAs. The most common mode of action of miRNAs is their interaction with the 3′ untranslated region (3′ UTR) of target mRNAs and increased mRNA degradation and translation suppression. miRNAs can also interact with the five UTR, coding sequence, and promoter regions of their target. In some cases, miRNA interaction with target sequences can induce transcription or regulate transcription. Various parameters modulate miRNA-mRNA interaction, including the subcellular state of miRNAs, the amount of miRNAs and target mRNAs, and the affinity of the interactions (Chen et al., 2015). miRNAs play a role in almost all aspects of cancer biology, such as apoptosis, proliferation, metastasis, and angiogenesis (Lee and Dutta, 2009). In addition, miRNAs have been proposed as potential biomarkers for the recognition of various different cancer types (Lin et al., 2015). Some studies also reported that several miRNAs have a potential value as diagnostic biomarkers of ovarian cancer (Banka and Dara, 2012; Yao et al., 2020).

### Related Works

The down-regulation of miRNAs was found to be related to the progression and the prognoses of cancers. Falzone et al. determined that a group of 16 miRNAs were significantly expressed between bladder cancer patients and normal samples; they serve to modulate the expression of both EMT and NGAL/MMP-9 pathways (Falzone et al., 2016). Falzone et al. identified a series of novel microRNAs and their diagnostic and prognostic significance in oral cancer and their study has therefore developed a molecular detector (Falzone et al., 2019). Another study by Asano et al. reported circulating serum miRNA profile classifier for the detection of sarcoma samples using seven miRNAs (Asano et al., 2019). Table 1 summarizes the results of miRNA associations with ovarian cancer in three recent genetic biomarker studies.

**TABLE 1:** Summary of miRNA genes shown to be statistically significantly associated with ovarian cancer.

Reference	Association	Up-regulated miRNA	Down-regulated miRNA
Tuncer et al. (2020)	Epithelial ovarian cancer	miR-6131, miR-1305, miR-197-3p, and miR-3651	miR-3135b, miR-4430, miR-664b-5p, and miR-766-3p
Nam et al. (2008)	Serous ovarian cancer	miR-16, miR-20a, miR-21, and miR-27a	miR-145, miR-125B, miR-125B, and miR-100
Iorio et al. (2007)	Epithelial ovarian cancer and normal	miR-200a, miR-141, miR-200c, miR-200b, miR-182, and miR-205	miR-127, miR-140, miR-9, miR-101, miR-147, miR-204, miR-211, miR-124a, and miR-302b

## Materials and Methods

### Candidate Genetic Biomarkers

To identify a robust circulating miRNA biomarker, we searched the Gene Expression Omnibus (GEO) database with specific keywords, namely, [“ovarian neoplasms” (MeSH Terms) OR ovarian cancer (All Fields)] AND “*Homo sapiens*” (porgn) AND [“microRNAs” (MeSH Terms) OR miRNA (All Fields)]. Then, two datasets using the same platform (3D-Gene Human miRNA V21_1.0.0) with larger sample sizes GSE106817 and GSE113486 were included (360 ovarian cancer patients and 2,811 non-cancer controls in total) for our analysis. GSE106817 (320 ovarian cancer patients and 2,759 non-cancer controls) was used as the internal discovery cohort, and GSE113486 (40 ovarian cancer patients and 52 non-cancer controls) was used for independent validation. This study was approved by the Ethics Committee of Tabriz University of Medical Sciences (No: IR. TBZMED.REC.1400.006).

### Data Preprocessing

**FIGURE 1:**
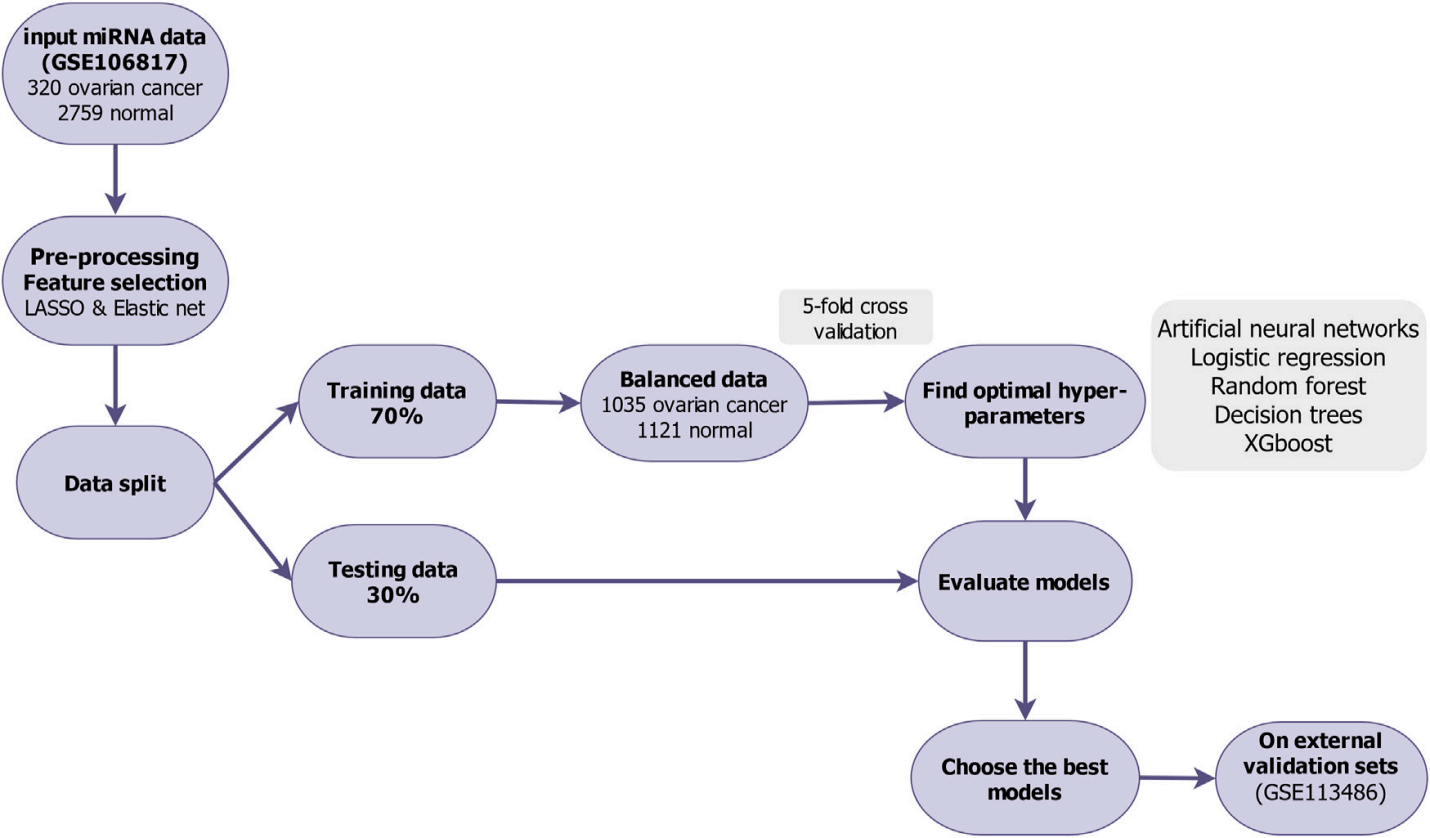
Flowchart of feature selection and model building in the study.

Our analytical process is summarized in Figure 1. To discover biomarkers for ovarian cancer, the free available dataset GSE106817 includes 320 ovarian cancer patients and 2,759 non-cancer controls (11% ovarian cancer and 89% non-cancer). For machine learning analysis purpose, we preprocessed, cleaned, and then normalized by min-max normalization the data (Huang J. et al., 2015).

### Feature Selection Algorithms

Feature (variable) selection is the main phase for selecting biomarkers in biological data with high dimension and small sample (p > n). Regularization is a kind of various technique of feature selection methods that use different penalty function to reduce the risk of overfitting and also reduce the complexity of the models (Drotár et al., 2015). Least Absolute Shrinkage and Selection Operation (LASSO) and Elastic Net are the most common embedded feature selection method which are an alternative to the subset selection and dimension reduction techniques. Thus, these algorithms can significantly reduce the variance by performing the variable selection. In the first phase, the expression levels of all 2,568 miRNAs from GSE106817 were analyzed to identify miRNAs as the candidate biomarkers by LASSO and Elastic Net (Zou and Hastie, 2005). For this sake, we used the “glmnet” package in R version 4.0.3. The next subsection gives a brief introduction to the LASSO and Elastic-Net.

#### LASSO

LASSO has been proposed by Tibshirani (Hastie et al., 2009) for parameter estimation and variable selection simultaneously in regression analysis. LASSO is a special instance of the penalized least squares regression with L1-penalty function. LASSO estimate of β can be defined as

$${\hat{\beta }}_{la}\left(\lambda \right)=\arg\min_{\beta }\left(\frac{\|Y-X{\beta \|}_{2}^{2}}{n}+\;\lambda {\left\|\beta \right\|}_{1}\right)\;;$$

Where

$${\left\|Y-X\beta \right\|}_{2}^{2}=\overset{n}{\underset{i=0}{\sum }}{\left(Y_{i}-\beta_{i}X_{i}\right)}^{2},\;{\left\|\beta \right\|}_{1}=\overset{k}{\underset{j=1}{\sum }}\left|\beta_{j}\right|\;\;\mathrm{and}\;\lambda \geq 0.$$

#### Elastic Net

Elastic Net (ENET) is a convex combination of Ridge and LASSO which shrinks some coefficients to be very small, and on the other hand, similar to the LASSO, ENET set some coefficients to be exactly zero. Elastic Net is an extension of the LASSO that is robust to extreme correlations among the predictors (Zou and Hastie, 2005). When the number of variables exceeds the number of instances (p > n), ENET performs better than LASSO. To trim the instability of the LASSO solution paths, when predictors are highly correlated, the Elastic Net was proposed for analyzing high dimensional data (Liang and Jacobucci, 2020). The Elastic Net uses a mixture of the LASSO and ridge regression penalties and can be formulated as:

$${\hat{\beta }}_{el}\left(\lambda \right)=\arg\min_{\beta }\left(\frac{\|Y-X{\beta \|}_{2}^{2}}{n}+\;\lambda_{2}{\left\|\beta \right\|}^{2}+\;\lambda_{1}{\left\|\beta \right\|}_{1}\right)\mathrm{and}\;\lambda_{1\;},\;\lambda_{2}\geq 0,\;\lambda_{1\;}+\lambda_{2}=\;1.$$

The entire path of variable selection by LASSO and ENET algorithms is computed by the path coordinate descent algorithms which is available “glmnet” package in R (Friedman et al., 2010).

### Machine Learning Classifier

Over the last decade, machine learning has been used for successful classification, both for identifying specific classes and for diagnosing cancers (Wang et al., 2005). We use this approach to characterize miRNAs with biomarker potential that could be useful for the diagnosis and/or prognosis of ovarian cancer for potential benefit for public health (screening) and for reduction in economic burden (Deb et al., 2018).

#### Logistic Regression

Logistic regression (LR) analyzes the relationship among multiple independent variables and a univariate binary outcome variable (Menard, 2010). One of the main advantage of the logistic regression is its simplicity and interpretability by providing the odds ratio for an outcome (Stoltzfus, 2011). The goodness of fit of a logistic regression model is evaluated using the area under the curve (AUC) (Abdulqader, 2017).

#### Artificial Neural Networks

Artificial neural networks (ANN) have been broadly used in medical studies (DeGregory et al., 2018). Such algorithms perform well when there are complex and non-linear associations between variables (Hassanipour et al., 2019). Briefly, artificial neural networks use predictors as inputs and connect them to multiple hidden layer combinations by assigning suitable weights to predict the outcome (Lisboa and Taktak, 2006). The hidden layers and weights must be appropriately selected by the analyst (Sherriff et al., 2004).

#### Decision Trees

Decision trees (DT) (Hassanipour et al., 2019) are a type of supervised machine learning that can be used to find attributes and extract patterns from big databases that are important for predictive modeling (Lisboa and Taktak, 2006). Decision trees are the most direct forward algorithm that processes a visual representation of the relationships between the independents and dependent variables (Hassanipour et al., 2019). However, the variation in the decision trees, in some instances, can be improved by using random forests for the outcomes of randomly generated decision trees to produce a more robust model (Vens et al., 2008).

#### Random Forest

Among several machine learning algorithms, random forest (RF) has a number of interesting characteristics. Firstly, RF does not overfit when the number of features exceeds the number of instances. Secondly, it does feature selection implicitly. Thirdly, it takes into account the interactions between variables (Okun and Priisalu, 2007). RF is an instance of ensemble learning, in which a complex model is made by combining many simple decision tree models to decrease the variance (Qi, 2012).

#### XGBoosting

XGBoost (XGB) abbreviated for extreme Gradient Boosting package. XGB is a decision-tree-based ensemble of machine learning algorithms that uses a scalable implementation of gradient boosting XGB framework tree boosting (Chen et al., 2015). The most significant component in XGB success is its scalability across all scenarios which is due to a number of major systems and algorithmic enhancements (Chen and Guestrin, 2016).

### Training Machine Learning Models and Hyper Parameter Setting

We started by removing the noise variables with LASSO and ENET. We then implemented SMOTE random oversampling techniques to balance cancer and non-cancer cases in the training data (GSE106817) using the “ROSE” package (Lunardon et al., 2014). We find the optimal prediction models in the training data by using 5-fold cross-validation. We performed ovarian cancer classification using ANN, LR, RF, DT, and XGB (James et al., 2013) algorithms to build our models, after finalizing the optimal hyperparameters for each model. The varImp () function in the *caret* package was used to determine the miRNAs that are the most important. In this, study we select the most important variables (variable importance >80%) from each of the models. We evaluated our model prediction performances based on several measures of accuracy, including sensitivity, specificity, area under the receiver operating characteristic (AUC), positive predictive value, negative predictive values, and Kappa (Collins et al., 2015). The ROC curves were analyzed by “pROC” in the R software.

Further, two online tools are applied to assess the biological plausibility of the selected miRNAs. To compare the microarray expression profiles of ovarian cancer to the non-cancer group, GEO2R is an interactive web tool that allows users to compare two or more groups of samples in a GEO Series. This procedure will enable the users to identify indicators that are differentially expressed across experimental conditions. To do this end, the limma R package implemented in GEO2R online tool, which generated adjusted *p*-value, B-statistic (or log-odds), Log2-fold change (logfc), and moderated t-statistic. MiRNet is an online tool for precision miRNA and xeno-miRNA analysis and functional interpretation. This tool contains a large amount of high-quality scientific data that connects miRNAs to their targets and other associated compounds (Fan et al., 2016).

## Results

GSE106817 included 2,568 miRNAs. Of those, LASSO and ENET identified 76 and 162 miRNAs, respectively. Then, the dataset was divided with a ratio of 70:30 for the training and testing set, respectively. For the training set, there were 2,156 samples and there were 923 samples in the testing set. The training set had 224 ovarian cancerous and 1,932 non-cancerous samples. After balancing the training data, the samples of non-cancerous decreased to 1,121 and cancerous samples increased to 1,035. Model fitting and tuning parameter selection by 5-fold cross-validation were done on the training data. The dataset with reduced features is classified using LR (statistical), DT and RF (tree-based), ANN and XGB (machine learning) classifier. In this study, the features with higher importance (over 80%) implemented in proposed models are shown in Table 2.

**TABLE 2:** miRNAs identified with threshold over 80% importance in both Lasso and Elastic net in the dataset GSE106817 with miRNA status.

miRNA-ID List	Importnace in Elastic Net	Importnace in LASSO (%)	adj.*p*-value	B	logFC	miRNAStatus
hsa-miR-5100	100	100	<0.001	16.18	4.15	Upregulated
hsa-miR-1290	100	100	<0.001	13.00	5.61	Upregulated
hsa-miR-320b	—	88.07	<0.001	12.25	4.11	Upregulated
hsa-miR-1233-5p	85.63	87.81	<0.001	11.78	2.36	Upregulated
hsa-miR-4783-3p	100	87.44	<0.001	10.36	2.89	Upregulated
hsa-miR-6800-5p	—	84.07	<0.001	8.66	−1.60	Downregulated
hsa-miR-4532	85.51	—	<0.001	6.95	2.90	Upregulated
hsa-miR-3184-5p	83.33	—	<0.001	5.29	−3.23	Downregulated
hsa-miR-4787-3p	100	—	<0.001	3.82	2.30	Upregulated
hsa-miR-1228-5p	88.83	—	<0.001	2.03	−0.93	Downregulated

We identified 10 potential miRNAs hsa-miR-5100, hsa-miR-6800-5p, hsa-miR-1233-5p, hsa-miR-4532, hsa-miR-4783-3p, hsa-miR-4787-3p, hsa-miR-1228-5p, hsa-miR-1290, hsa-miR-3184-5p, and hsa-miR-320b from the GSE106817 datasets and were defined as the candidate miRNAs for ovarian cancer diagnosis. It is clear that hsa-miR-1233-5p, hsa-miR-4783-3p, hsa-miR-5100, and hsa-miR-1290 are features identified by both feature selection methods. hsa-miR-320b and hsa-miR-6800-5p have been identified as important features by LASSO, and hsa-miR-4532, hsa-miR-3184-5p, hsa-miR-4787-3p, and hsa-miR-1228-5p have been recognized by ENET.

The results of GEO2R (generated by the limma) are presented in Table function (Table 2). Note that the column of adjusted *p*-value is generally recommended as the primary statistic in the interpretation of results. The miRNAs with the smallest *p*-values will be the most reliable, and column B shows that the represented miRNAs are differentially expressed and logfc presented change between normal and cancerous conditions. As shown in Table 2, all upregulated miRNAs have logfc > 2 and all of miRNAs have adjusted *p*-value <0.0001. Based on the 10 selected miRNAs, the final machine learning models with optimal hyperparameters are presented in Table 3.

**TABLE 3:** Predictive power of models for ovarian cancer classification and prediction in the external (GSE113486) validation data.

Classifier	Hyperparameters	AUC^a^ (%)	Accuracy (%)	Sensitivity (%)	Specificity (%)	Negative predictive value (%)	Positive predictive value (%)	Kappa (%)
LR	Parameters^b^	100	100	100	100	100	100	100
DT	Cp^c^ = 0.0115942	92.60	91.30	92.50	90.38	88.10	94	82.41
RF	Mtry^d^ = 2	100	97.83	95	100	100	96.30	95.55
ANN	Size^e^ = 3 and decay^f^ = 1e−04	100	100	100	100	100	100	100
XGB	nrounds = 50, max_depth^g^ = 2, eta = 0.3, gamma^h^ = 0, colsample_bytree^i^ = 0.8, min_child_weight^j^ = 1 and subsample^k^ = 1	100	98.91	97.50	100	100	98.11	97.78

a The area under the receiver operating characteristic curve (maximum) was used to select the optimal model.

^b^The formula for logistic regression for prediction of ovarian cancer is p = (1 + e^−[14.19−40.34(has.miR.6800.5p)+3.61(has.miR.1228.5p)+16.09(has.miR.5100)+2.86(has.miR.1290)+4.17(has.miR.4783.3p)−8.9(has.miR.3184.5p)+8(has.miR.320b)+9.23(has.miR.4532)−4.2(has.miR.4787.3p)−0.65(has.miR.1233.5p)]^)^−1^.

c The complexity parameter (cp) is used to control the size of the decision tree and to select the optimal tree size. If the cost of adding an additional variable to the decision tree from the current node is above the value of the cp, then tree building does not continue.

d mtry is the number of variables available for splitting at each tree node. In the random forests literature, this is referred to as the mtry parameter.

e Size is the number of units in a hidden layer.

f Decay is the regularization parameter used to avoid over-fitting.

g max-depth is used to control over-fitting as higher depth will allow model to learn relations very specific to a particular sample.

h gamma A node is split only when the resulting split gives a positive reduction in the loss function. Gamma specifies the minimum loss reduction required to make a split. Makes the algorithm conservative. The values can vary depending on the loss function and should be tuned.

i Denotes the fraction of columns to be randomly sampled for each tree.

j min_child_weight used to control over-fitting. Higher values prevent a model from learning relations which might be highly specific to the particular sample selected for a tree. Too high values can lead to under-fitting; hence, it should be tuned using CV.

k Subsample lower values make the algorithm more conservative and prevent overfitting, but too small values might lead to under-fitting.

**FIGURE 2:**
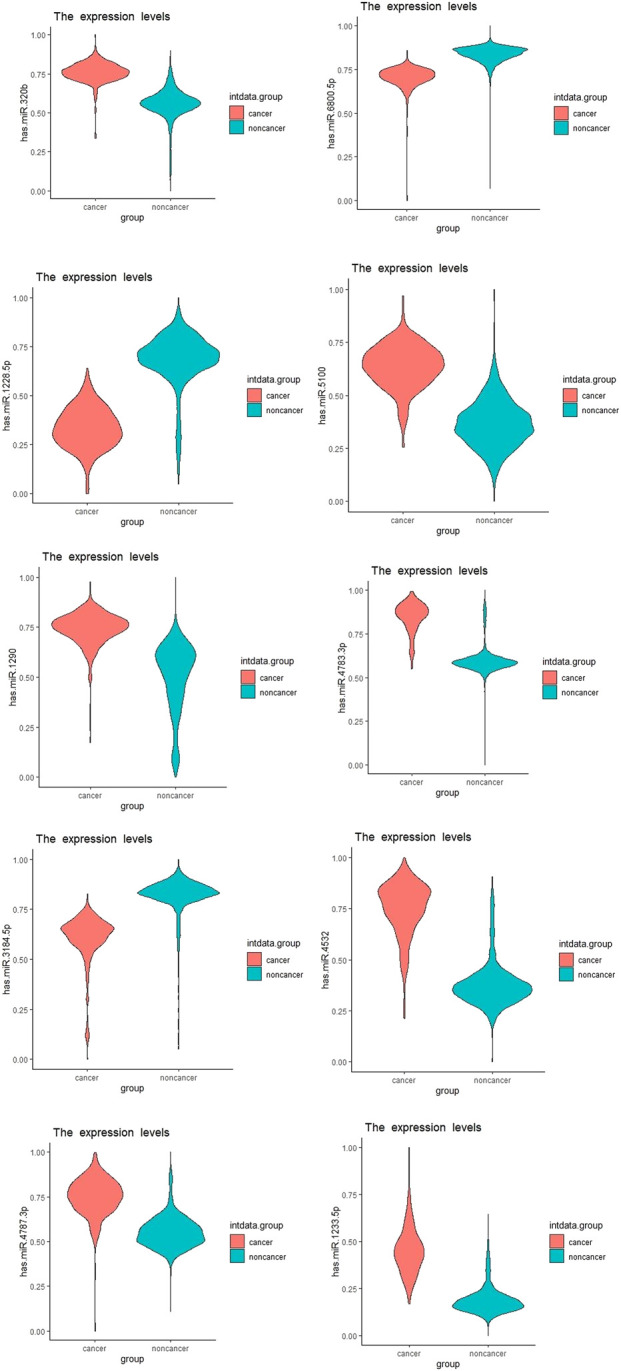
Boxplots of the 10 identified miRNAs in ovarian cancer patients compared with the non-cancer control patients in the dataset GSE106817.

**FIGURE 3:**
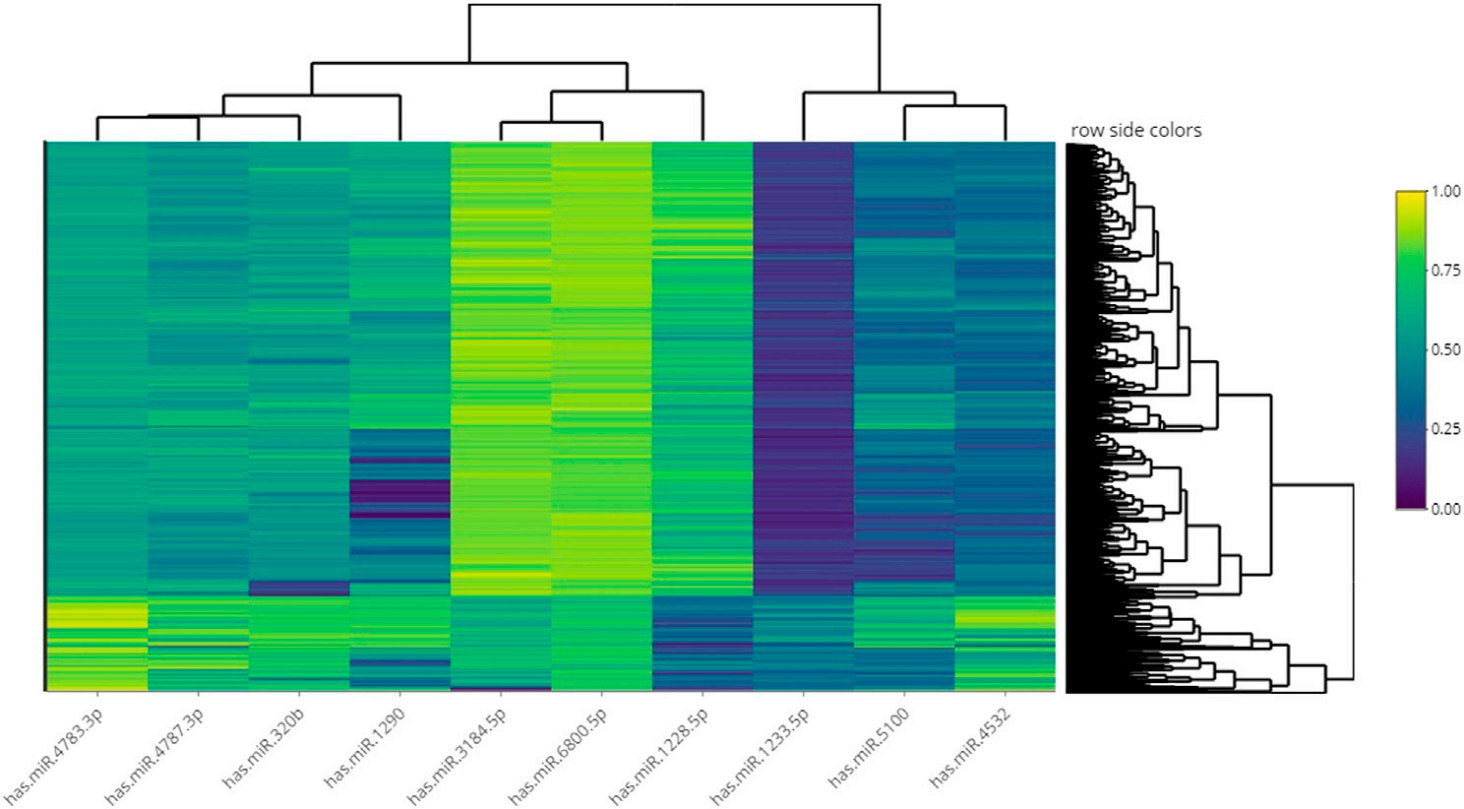
Heatmap of hierarchical clustering analysis using the 10 identified miRNAs to distinguish different samples in the dataset GSE106817.

**FIGURE 4:**
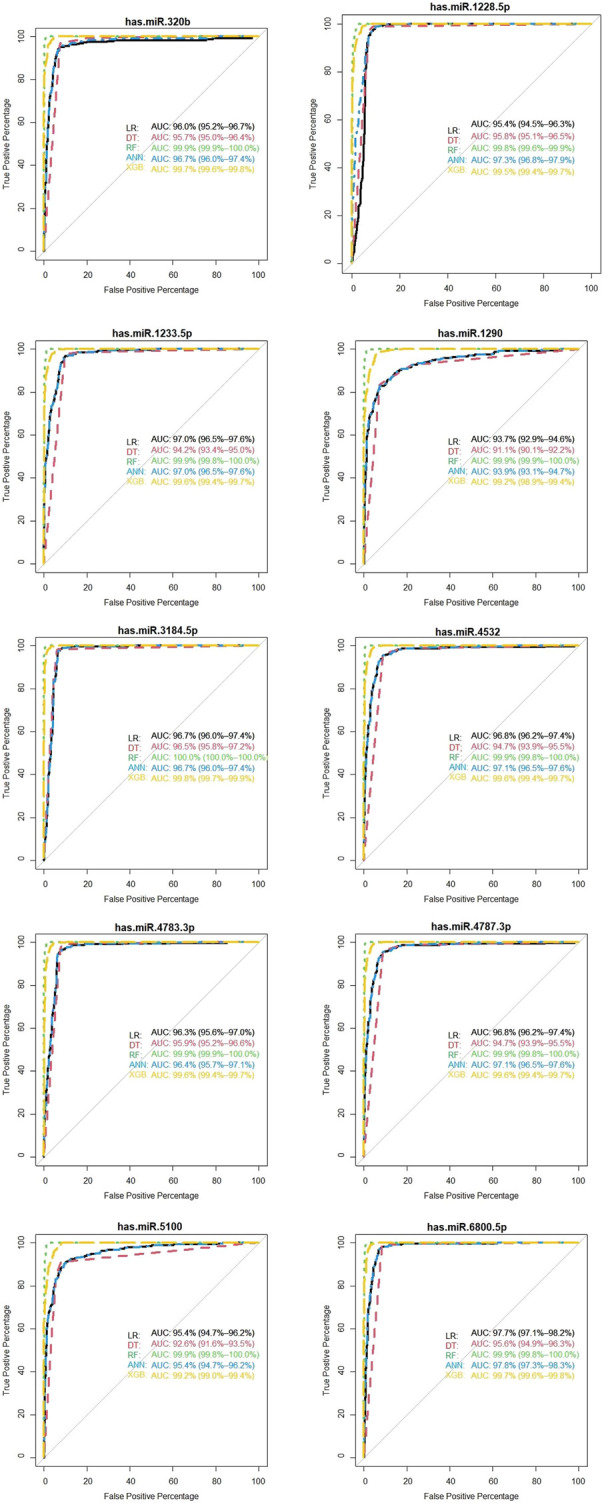
Diagnostic performance of the 10 identified serum miRNA signatures in the internal (GSE106817) data.

**FIGURE 5:**
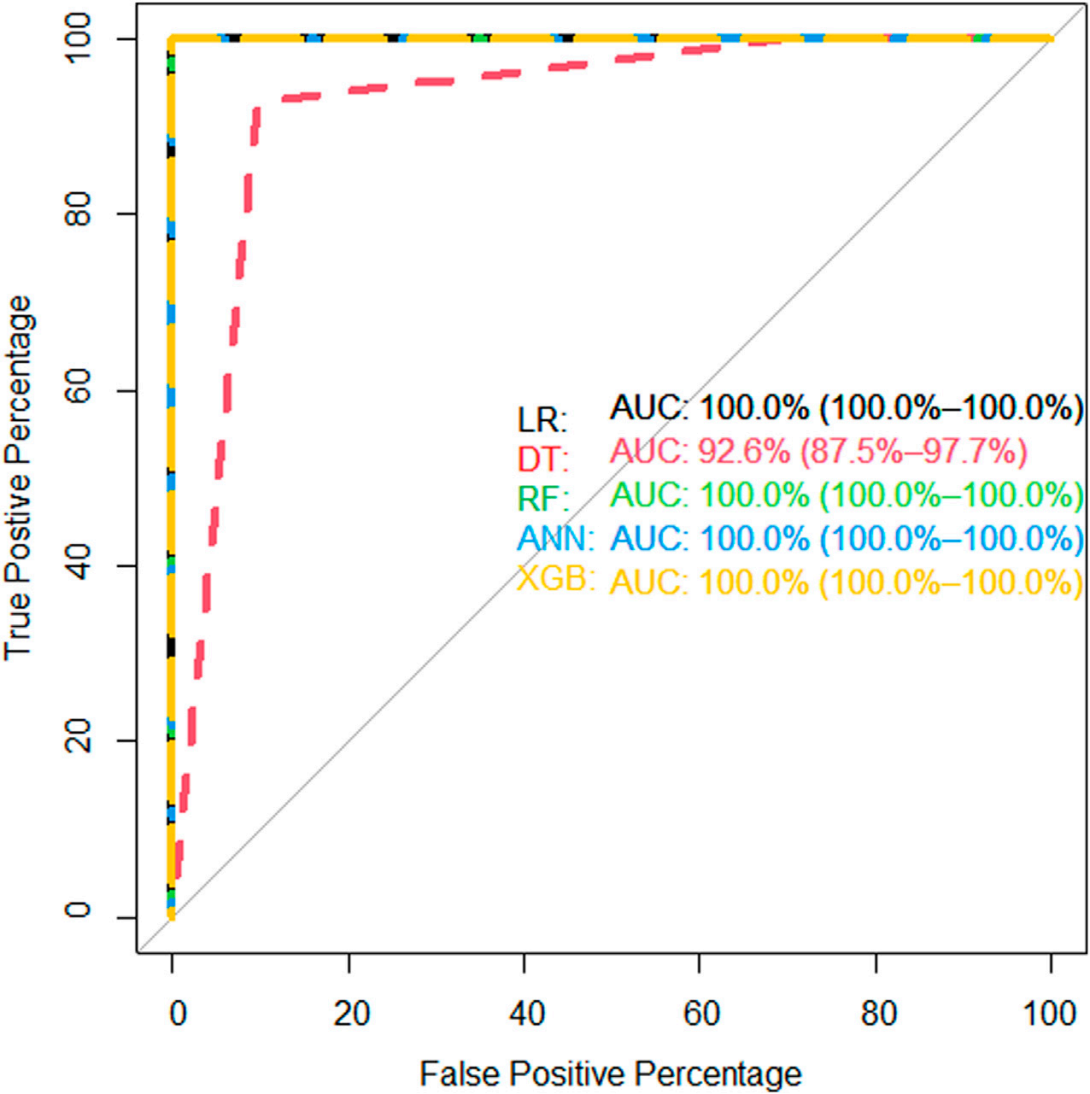
AUC of proposed models of all identified microRNAs in the internal (GSE106817) validation data.

**FIGURE 6:**
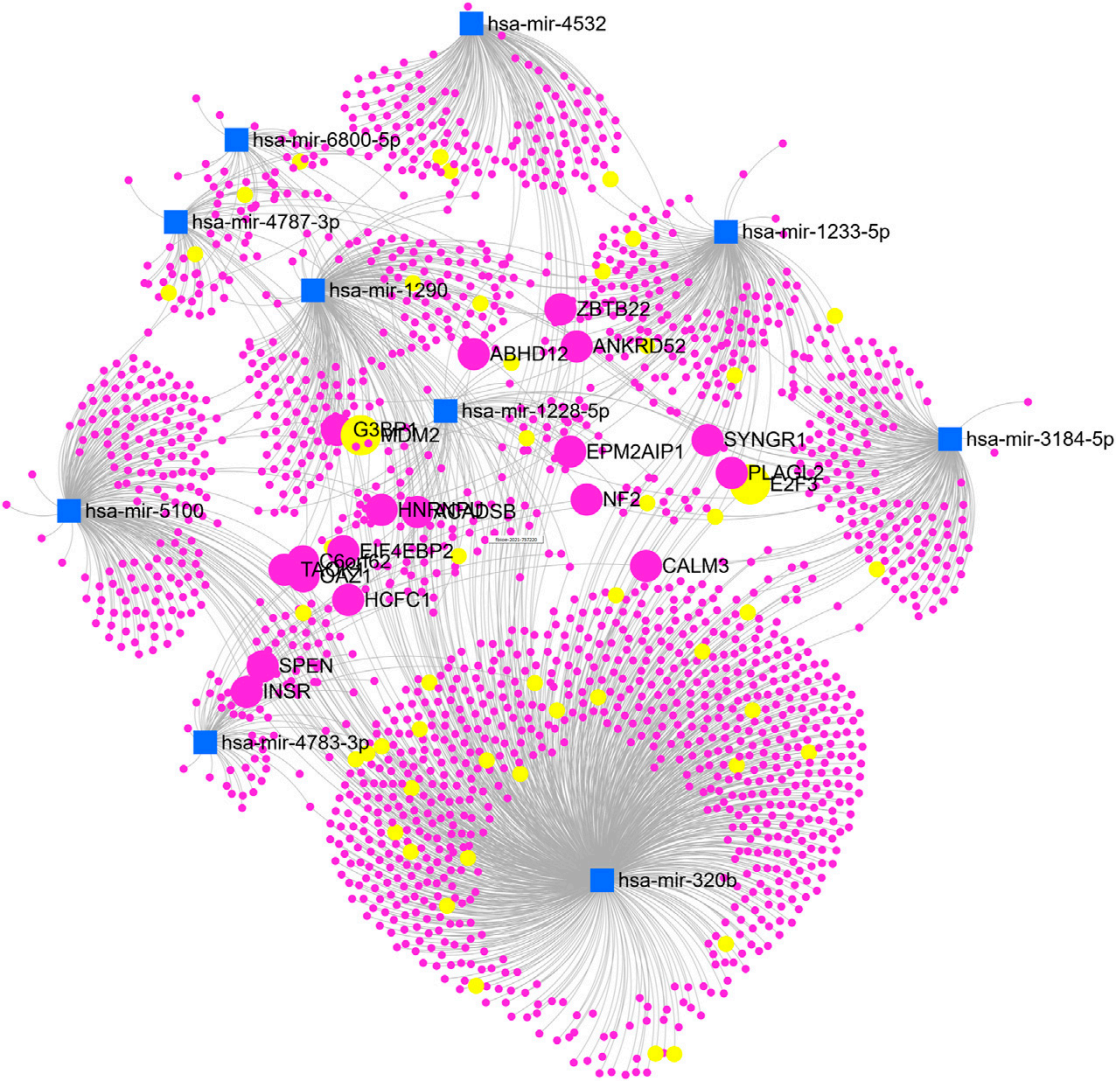
The miRNA network with target genes.

We showed the expression levels of these 10 identified miRNAs in the internal datasets using a boxplot (Figure 2); among them, seven miRNAs (hsa-miR-320b, hsa-miR-5100, hsa-miR-4783-3p, hsa-miR-1290, hsa-miR-4532, hsa-miR-4787-3p, and hsa-miR-1233-5p) identified the most significantly up-regulated in ovarian cancer samples compared to non-cancer samples. The heatmap using the “pheatmap” package shows differences between samples in each group. In Figure 3 (the heatmap of GSE106817), the miRNAs has-mir-3184-5p, has-mir-6800-5p, and has-mir-1228-5p in the left hand side of the figure show a significantly low expression level in the ovarian cancer group (red color). However, hsa-mir-5100, hsa-mir-1290, hsa-mir-320b, hsa-mir-1233-5p, hsa-mir-4532, hsa-mir-4783-3p, and hsa-mir-4783-3p have the high expression levels in the cancerous group (light yellow color). The individual AUCs of these 10 identified miRNAs are listed in Figure 4 which shows that each of 10 miRNAs has high AUC in all proposed models. Next, AUCs of all selected miRNAs are presented in Figure 5 which clearly indicates that all moles, except DT, have above 99% AUC. All miRNA-target gene interactions are represented in Figure 6. The purple circles represent the target genes implicated in cancer-related pathways that are shown by yellow circles.

### Model Evaluation in External Validation Data

Given the robust performance of 10 miRNAs in the internal datasets, we further examined their performance in independent external validation (GSE113486). External validation dataset (GSE113486) has 40 ovarian cancer patients and 52 non-cancer controls (43% ovarian cancer, 57% non-cancer). We found that all the miRNAs had high performance and could efficiently distinguish the ovarian cancer samples from non-cancer controls.

**FIGURE 7:**
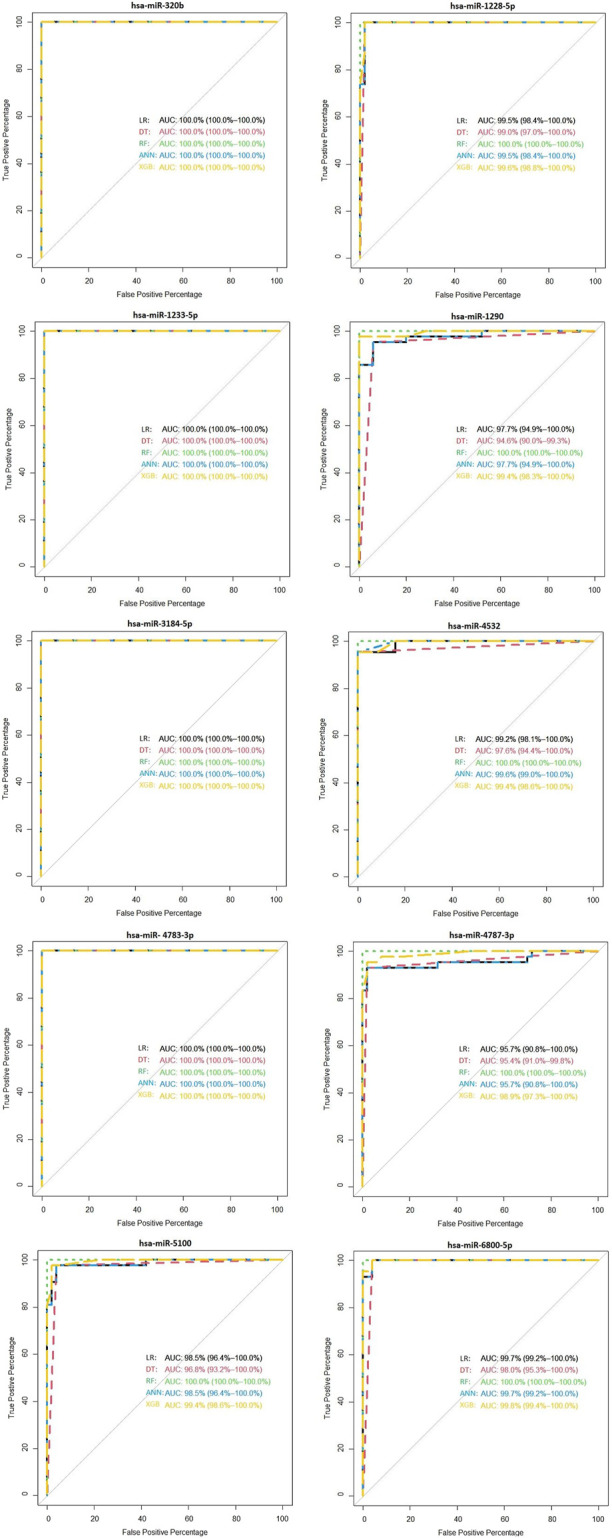
Validation of the diagnostic performance of the three selected miRNA high signatures in the external (GSE113486) data.

As shown in Figure 7, hsa-miR-320b, hsa-miR-1233-5p, hsa-miR-3184-5p, and hsa-miR-4783-3p have 100% of AUC in all proposed models. In the external validation dataset (GSE113486), the AUC of each candidate miRNAs was over 95% (minimum AUC: 95.7%, maximum AUC: 100%) for ovarian cancer classification (Figure 7). From Supplementary Figure S2, it is clear that, except DT, other machine learning models have an AUC over 100% in the external validation dataset with 10 selected miRNAs.

The models that yielded the highest AUC, accuracy, and sensitivity are shown in Table 3. As displayed in Table 3 (and also Supplementary Figure S2), we found four models yielded 100% AUC; however, DT did not have a strong performance because it is weak learner (Drucker and Cortes, 1996).

Finally, to make use of our prediction models, the practitioners can give the values of the 10 selected miRNAs in the online excel sheet (https://ufile.io/t2exrfph) and calculate the probability of the ovarian cancer for the patient (Supplementary Figure S1).

## Discussion

In the early phases, ovarian cancer is mostly asymptomatic or existent with only non-specific symptoms (Desai et al., 2014; Tuncer et al., 2020). Intervention at this phase makes ovarian cancer almost curable, and thus, early detection and diagnosis are critical to decrease the incidence and mortality of ovarian cancer (Zhang et al., 2011). Therefore, in this study, we used effective strategies and identified 10 miRNAs (hsa-miR-5100, hsa-miR-6800-5p, hsa-miR-1233-5p, hsa-miR-4532, hsa-miR-4783-3p, hsa-miR-4787-3p, hsa-miR-1228-5p, hsa-miR-1290, hsa-miR-3184-5p, and hsa-miR-320b) as strong potential biomarkers for ovarian cancer. We found that these miRNAs (all together) had high enough prediction accuracy for identification of ovarian cancer from non-cancer (logistic regression had an AUC 100%, sensitivity 100%, and specificity 100%; decision trees had an AUC 92.60%, sensitivity 92.5%, and specificity 90.38%; random forest had an AUC 100%, sensitivity 95%, and specificity 100%; artificial neural network had an AUC 100%, sensitivity 100%, and specificity 100.0%; and XGBoost had an AUC 100%, sensitivity 97.50%, and specificity 100%). Furthermore, hsa-miR-5100, hsa-miR.4532, hsa-miR.4783.3p, and hsa-miR-320b were more stable in the discovery and validation datasets.

### Biological Insight

There is evidence in the literature for the biomarkers included in our study. Huang et al. (2011) showed that modulation of miR-5100 could potentially be employed as a therapeutic target for cancer (Huang H. et al., 2015). It has shown that major target gene of miR-5100 is AZIN1. AZIN1 gene encodes antizyme inhibitor 1, the first member of this gene family that is ubiquitously expressed, and is localized in the nucleus and cytoplasm. Overexpression of antizyme inhibitor one gene has been associated with increased proliferation, cellular transformation, and tumorigenesis (Hu et al., 2017). Also, our result is important about the relationship between ovarian cancer and miR-5100 because of target gene function. Tuncer et al. (2020) suggested that hsa-miR-6800-5p is an effective biomarker for ovarian cancer. MiR-1233 is considered an oncomiRNA since it targets p53, inhibiting its function in RCC (Iwamoto et al., 2014). Hu et al., (2017) showed that miR-4532 is involved in the multidrug resistance formation in breast cancer by targeting hypermethylated cancer 1 (*HIC*-*1*), a tumor-suppressor gene (Feng et al., 2018). Also, hsa-miR-4783-3p has a major target of INSM1/IA-1 (insulinoma-associated one gene) (http://mirdb.org/) and this gene is a developmentally regulated zinc-finger transcription factor, exclusively expressed in the foetal pancreas and nervous systems, and in tumours of neuroendocrine origin (Juhlin et al., 2020). Li et al., 2016 suggest that miRNA-1228 is deregulated, and the most encompassed biological pathways are apoptosis-related (Li et al., 2016). In another study, miR-1290 is significantly overexpressed in patients with high-grade serous ovarian carcinoma (HGSOC) and they suggested that it is a new potential diagnostic biomarker for HGSOC. Exosomal miR-1290 is a potential biomarker of high-grade serious ovarian carcinoma (Cortez et al., 2018). The study of Tuncer et al. (2020) revealed that miR-320b belonged to the miR-320 family which has low expression levels in ovarian cancer. Prior studies indicated that decreased expression level of the miR-320 family is associated to activate cell proliferation (Tuncer et al., 2020). We have analyzed the major target genes of the upregulated miRNA interactions (Supplementary Figure S3). We found only two gene interactions with string database system, especially TP53 and HIC1 genes associated with a related system in human metabolism (Supplementary Figure S3).

### Strengths and Limitations

This study has several strengths. Firstly, we applied logistic regression and four of the main machine learning approaches to predict ovarian cancer. Secondly, we identified predictive models to predict the ovarian cancer. Our findings provided strong evidence that the serum miRNA profile represented a promising diagnostic biomarker for ovarian cancer. Thirdly, we used two robust variable selection approaches to identify the important miRNAs. Finally, we evaluated the prediction accuracy of the proposed prediction models in both internal and external data to provide more robust results for practical and clinical applications.

However, there were certain limitations in our study. We had relatively small sample size in ovarian cancer group. Other limitations were the pathological information such as the tumor stage, age, or other factors which were not available in GSE106817 dataset. Nonetheless, the prediction accuracy of our model has high enough (100% AUC) for clinical use. But we still suggest further study to consider age, stage, and other unrecognized factors associated with ovarian cancer that has not included in the current paper. Also, we restricted our analysis to ovarian cancer patients and non-cancer controls, and we did not evaluate the capability of these miRNAs to distinguish ovarian cancer from other cancers.

## Conclusion

In this paper, we used the state-of-the-art machine learning algorithms along with so-called penalized statistical approaches to model ovarian cancer with miRNA data. Our algorithms selected 10 important miRNA that can predict the ovarian cancer with an AUC of 100%. Our findings provided significant evidence that the serum miRNA profile represents a promising diagnostic biomarker for ovarian cancer.

## Data Availability

The original contributions presented in the study are included in the article/Supplementary Material. Further inquiries can be directed to the corresponding author.
