# Supplementary material for: Exploration of Potential miRNA Biomarkers and Prediction for Ovarian Cancer Using Artificial Intelligence
Source: Front Genet. 2021 Nov 25;12:724785. doi: 10.3389/fgene.2021.724785 (PMC8656459; doi:10.3389/fgene.2021.724785)
Supplement: Supplementary file 1 [file DataSheet1.docx]

Supplementary Table 1. Optimal hyperparameters in proposed methods (in training set of internal datasets)

| **Method** | **Optimal hyperparameters in training sample ^1^** | **Sensitivity** | **Specificity** | **Area under the receiver operating characteristic curve** |
| --- | --- | --- | --- | --- |
| **Logistic regression** | **-** | **98.30%** | **99.03%** | **99.81%** |
| **Decision trees** | **Cp^2^ = 0.0115942** | **97.77%** | **97.97%** | **98.56%** |
| **Random forests** | **Mtry^3^ = 2** | **99.19%** | **100%** | **100%** |
| **Artificial neural networks** | **Size^4^ = 3 and decay^5^ = 1e-04** | **99.55%** | **99.86%** | **99..99%** |
| **XGBoost** | **nrounds = 50, max_depth^6^ = 2, eta = 0.3, gamma^7^ = 0, colsample_bytree^8^ = 0.8, min_child_weight^9^ = 1 and subsample^10^ = 1** | **99.46%** | **-** | **99.99%** |

^1^The area under the receiver operating characteristic curve (maximum) was used to select the optimal model. ^2^The complexity parameter (cp) is used to control the size of the decision tree and to select the optimal tree size. If the cost of adding an additional variable to the decision tree from the current node is above the value of the cp, then tree building does not continue. ^3^mtry is the number of variables available for splitting at each tree node. In the random forests literature, this is referred to as the mtry parameter. ^4^Size is the number of units in a hidden layer. ^5^Decay is the regularization parameter used to avoid over-fitting. ^6^max-depth used to control over-fitting as higher depth will allow model to learn relations very specific to a particular sample. ^7^gamma A node is split only when the resulting split gives a positive reduction in the loss function. Gamma specifies the minimum loss reduction required to make a split. Makes the algorithm conservative. The values can vary depending on the loss function and should be tuned. ^8^ Denotes the fraction of columns to be randomly samples for each tree. ^9^ min_child_weight used to control over-fitting. Higher values prevent a model from learning relations which might be highly specific to the particular sample selected for a tree. Too high values can lead to under-fitting hence, it should be tuned using CV. ^10^ subsample lower values make the algorithm more conservative and prevents overfitting but too small values might lead to under-fitting.

| **Classifier** | **AUC(%)** | **Accuracy(%)** | **Senisitivity(%)** | **Specifitiy(%)** | **Negative predictive value(%)** | **Positive**  **predictive value(%)** | **Kappa(%)** |
| --- | --- | --- | --- | --- | --- | --- | --- |
| **LR** | 99.5 | 98.05 | 97.92 | 98.07 | 99.75 | 85.45 | 98.28 |
| **DT** | 95.7 | 97.4 | 94.79 | 97.73 | 99.38 | 82.72 | 86.89 |
| **RF** | 100 | 99.02 | 97.92 | 99.15 | 99.76 | 93.07 | 94.89 |
| **ANN** | 99.9 | 97.83 | 96.88 | 97.94 | 99.63 | 84.55 | 89.08 |
| **XGB** | 99.9 | 98.37 | 96.88 | 98.55 | 99.63 | 88.57 | 91.63 |

Supplementary Table 2. Predictive power of models for ovarian cancer classification and prediction in the internal validation dataset GSE106817.

| **Classifier** | **AUC(%)** | **Accuracy(%)** | **Senisitivity(%)** | **Specifitiy(%)** | **Negative predictive value(%)** | **Positive**  **predictive value(%)** | **Kappa(%)** |
| --- | --- | --- | --- | --- | --- | --- | --- |
| **LR** | 100 | 100 | 100 | 100 | 100 | 100 | 100 |
| **DT** | 92.60 | 91.30 | 92.50 | 90.38 | 88.10 | 94 | 82.41 |
| **RF** | 100 | 97.83 | 95 | 100 | 100 | 96.30 | 95.55 |
| **ANN** | 100 | 100 | 100 | 100 | 100 | 100 | 100 |
| **XGB** | 100 | 98.91 | 97.50 | 100 | 100 | 98.11 | 97.78 |

Supplementary Table3. Predictive power of models for ovarian cancer classification and prediction in the external validation dataset GSE113486.


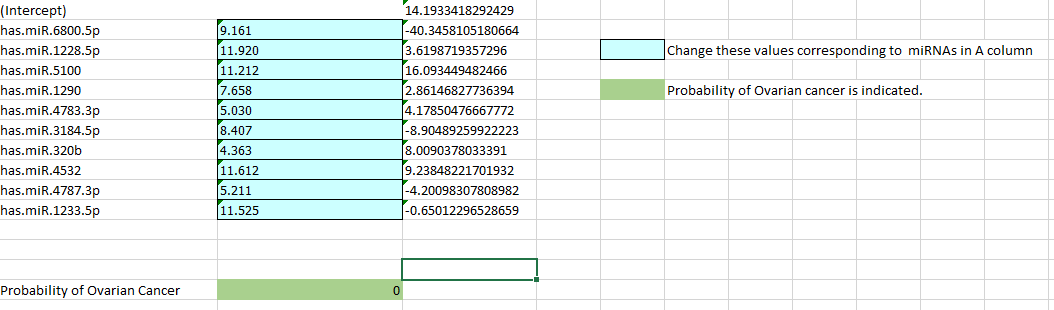


Supplementary Figure 1. Online tool for calculation the probability of the ovarian cancer.


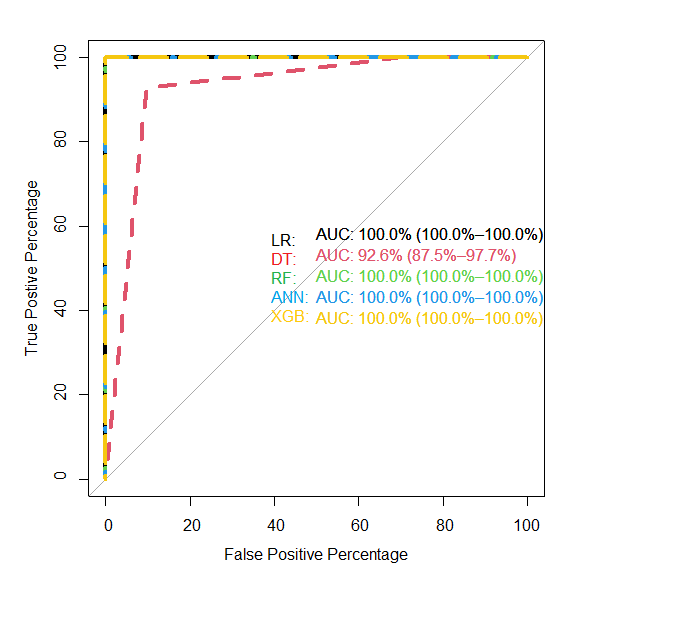


Supplementary Figure 2. AUC of proposed models of all identified microRNAs in the external (GSE113486) validation data.


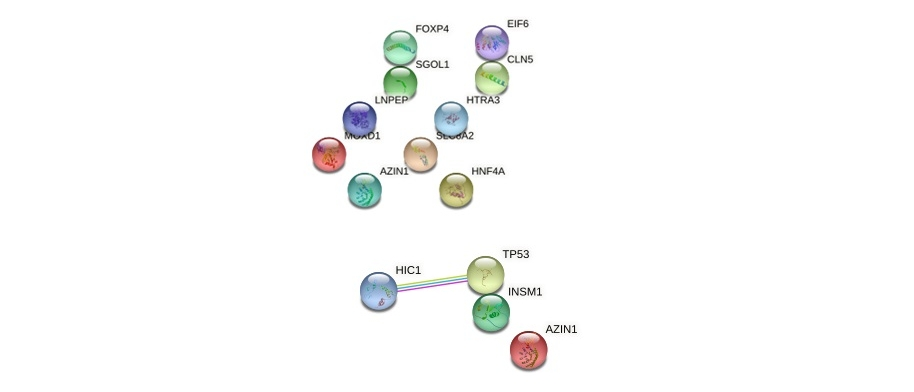


Supplementary Figure 3. Major target genes of upregulated miRNAs with string database analysis.
